# Author Correction: *Tiller Number1* encodes an ankyrin repeat protein that controls tillering in bread wheat

**DOI:** 10.1038/s41467-023-39365-w

**Published:** 2023-06-14

**Authors:** Chunhao Dong, Lichao Zhang, Qiang Zhang, Yuxin Yang, Danping Li, Zhencheng Xie, Guoqing Cui, Yaoyu Chen, Lifen Wu, Zhan Li, Guoxiang Liu, Xueying Zhang, Cuimei Liu, Jinfang Chu, Guangyao Zhao, Chuan Xia, Jizeng Jia, Jiaqiang Sun, Xiuying Kong, Xu Liu

**Affiliations:** 1 State Key Laboratory of Crop Gene Resources and Breeding, Institute of Crop Sciences, Chinese Academy of Agricultural Sciences, Beijing, 100081 China; 2 State Key Lab of Rice Biology, China National Rice Research Institute, Hangzhou, 310006 China; 3 National Centre for Plant Gene Research (Beijing), Innovation Academy for Seed Design, Institute of Genetics and Developmental Biology, Chinese Academy of Sciences, Beijing, 100101 China; 4 College of Advanced Agricultural Sciences, University of Chinese Academy of Sciences, Beijing, 100049 China

**Keywords:** Shoot apical meristem, Plant breeding, Agricultural genetics, Development

Correction to: *Nature Communications* 10.1038/s41467-023-36271-z, published online 14 February 2023

The original version of this Article contained an error in Fig. 6b, in which the two labels “TN1^ANK^-GFP” and “TaPYL-1D-Flag” in the top-left corner of the display were swapped. This has been corrected in both the PDF and HTML versions of the Article.

