# Supplementary material for: Tiller Number1 encodes an ankyrin repeat protein that controls tillering in bread wheat
Source: Nat Commun. 2023 Feb 14;14:836. doi: 10.1038/s41467-023-36271-z (PMC9929037; doi:10.1038/s41467-023-36271-z)
Supplement: Supplementary file 1 — Supplementary Information [file 41467_2023_36271_MOESM1_ESM.pdf]

Tiller Number1 encodes an ankyrin repeat protein that controls tillering in bread wheat

Dong and Zhang *et al.*

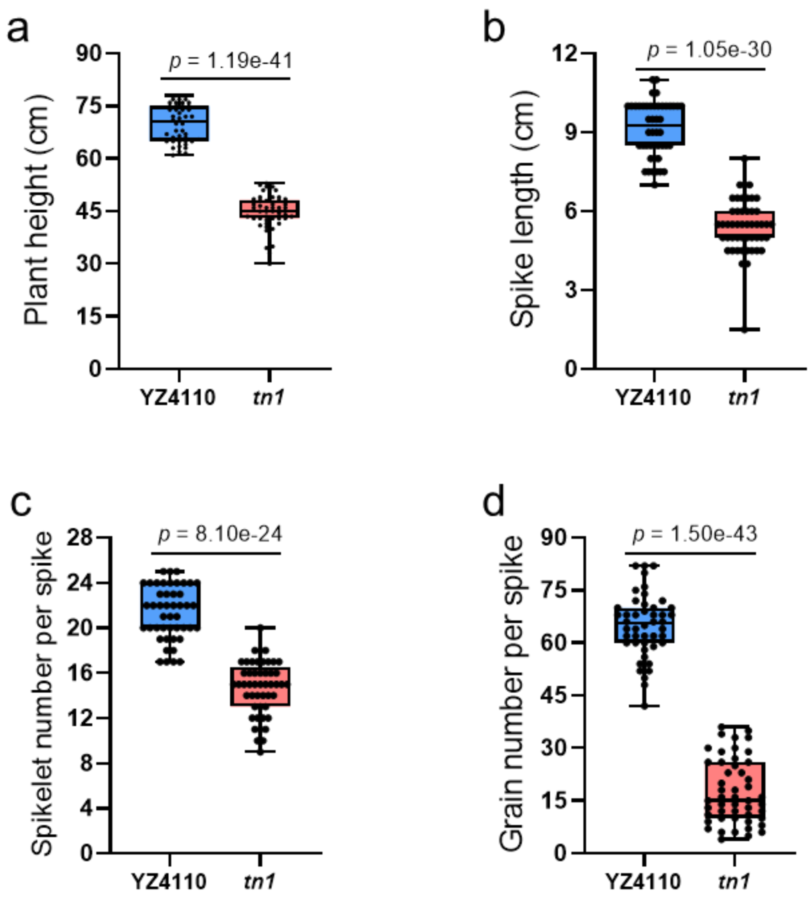

Supplementary Fig. 1. Major agronomic traits in YZ4110 and the *tn1* mutant.

a–d, Plant height (**a**), spike length (**b**), spikelet number per spike (**c**), and grain number per spike (**d**) of YZ4110 (n = 46 biologically independent samples) and the *tn1* mutant (n = 49 biologically independent samples). Data are means ± SD, and p values are indicated by two-tailed unpaired t-test. The two whiskers of the box plot and the middle, upper, and lower box lines represent the maximum, minimum, median and two quartiles of values in each group. Source data are provided as a Source Data file.

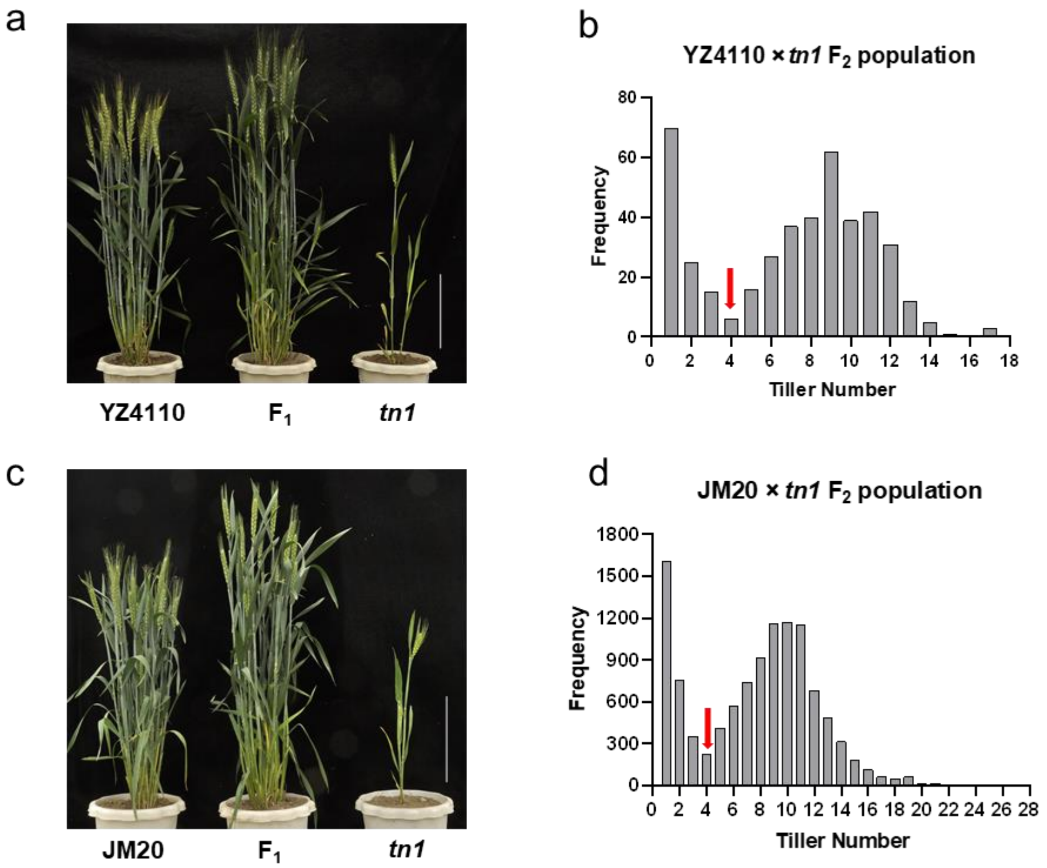

Supplementary Fig. 2. Genetic analysis of the *tn1* mutant.

a, Morphology of YZ4110, *tn1*, and the resulting F_1 plants. **b,** Frequency distribution of tiller number in the F_2 population derived from the cross between YZ4110 and the *tn1* mutant. **c,** Morphology of JM20, *tn1*, and the resulting F_1 plants. **d,** Frequency distribution of tiller number in the F_2 population derived from the cross between JM20 and the *tn1* mutant. Scale bars, 20 cm in (**a**) and (**c**). The red arrows in (**b**) and (**d**) indicate the trait value boundary. Source data are provided as a Source Data file.

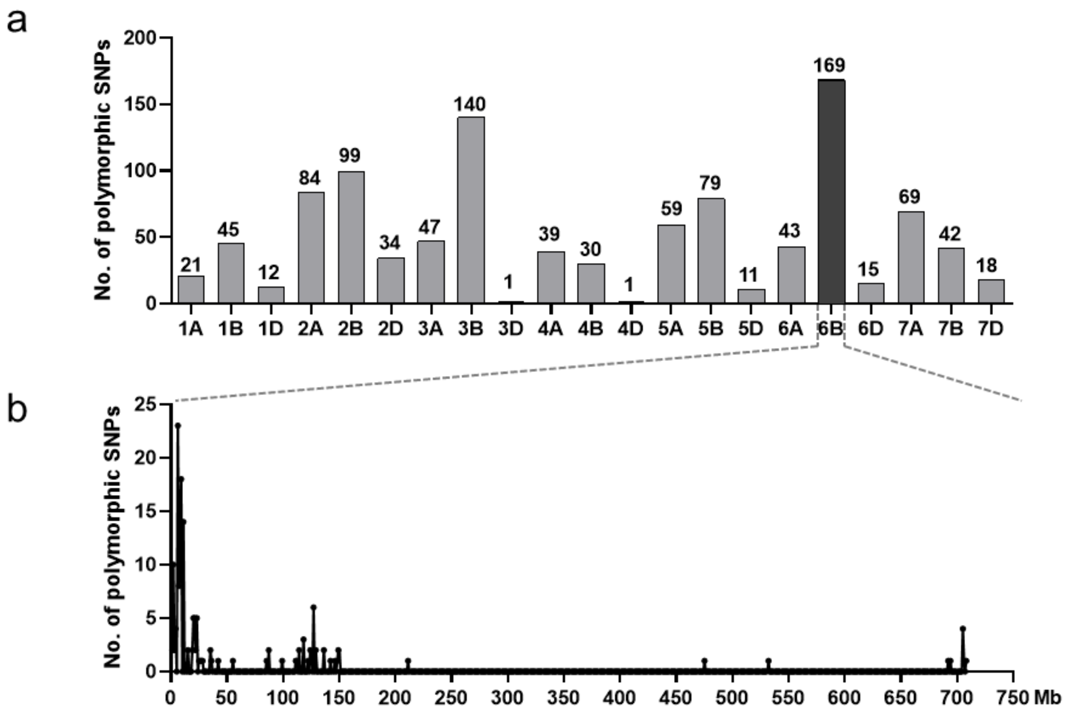

Supplementary Fig. 3. Distribution of single-nucleotide polymorphisms (SNPs) with biased frequency between pools based on wheat 660K SNP arrays.

a, Number of SNPs with biased frequency on each of the 21 wheat chromosomes based on the wheat 660K SNP array. **b,** Distribution of the SNPs on chromosome 6B. Source data are provided as a Source Data file.

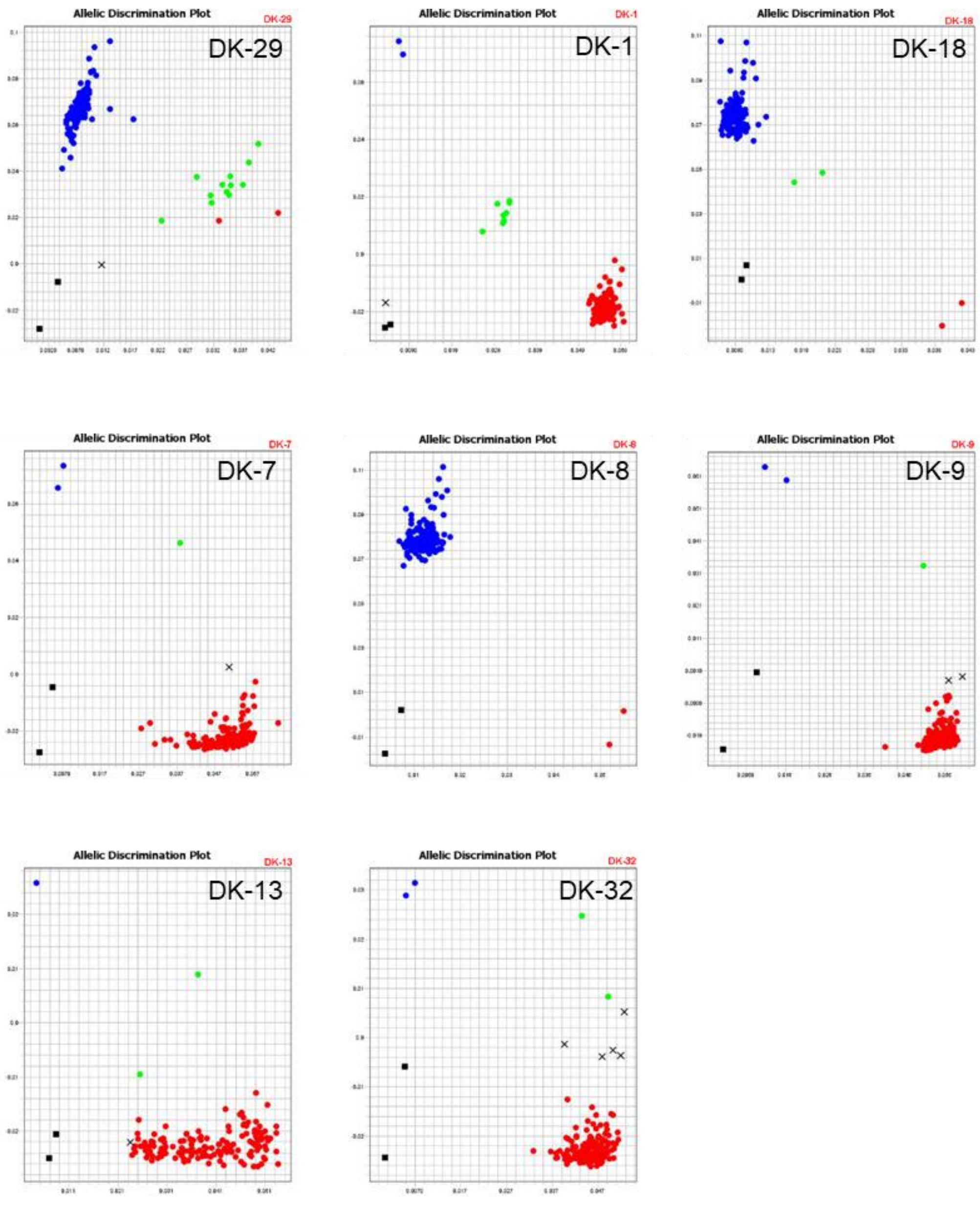

Supplementary Fig. 4. Allelic discrimination plot of kompetitive allele-specific PCR markers in primary mapping of *TN1*.

Genotyping analysis of the 186 recessive F_2 individuals, JM20, and *tn1* mutants in primary mapping of *TN1*. Each dot represents one sample; the black squares and green dots represent the non-template control and heterozygous alleles, respectively. The different homozygous alleles are represented by blue and red dots. The black crosses indicate the samples with undetermined genotype.

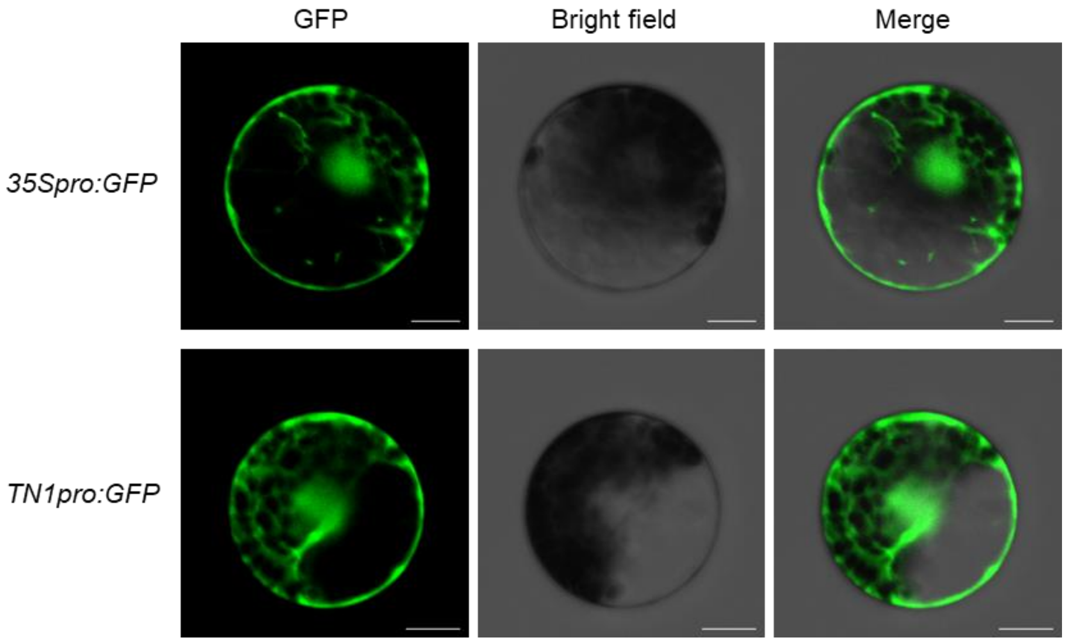

Supplementary Fig. 5. Activity test of the *TN1* promoter.

The *TN1pro:GFP* construct, containing a 3,251-bp putative promoter of the *TN1* gene, was transfected into wheat leaf mesophyll protoplasts by the PEG-mediated method. Scale bars, 10 μm. Images are representative of three independent experiments.

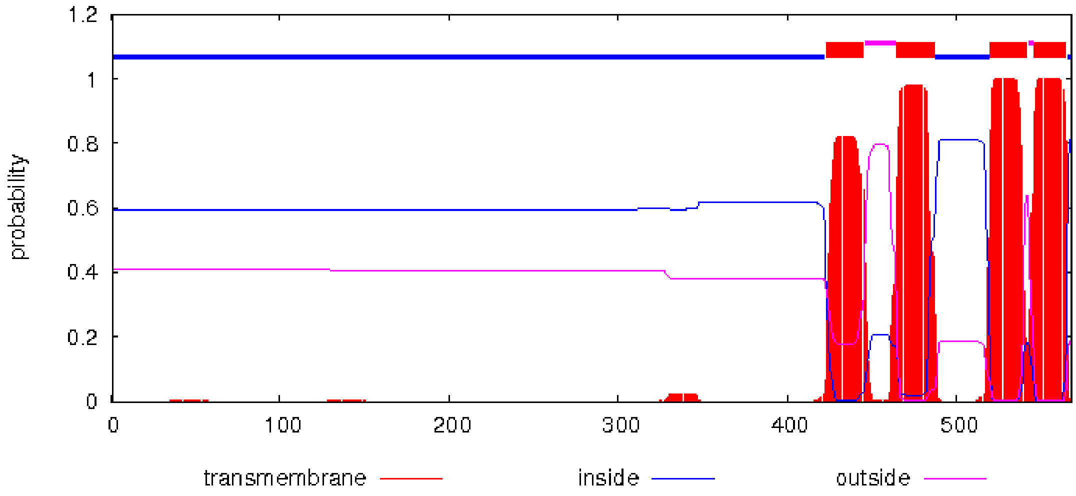

Supplementary Fig. 6. Topology prediction of the TN1 protein.

Topology prediction for TN1 using a transmembrane domain hidden Markov model (TMHMM version 2.0).

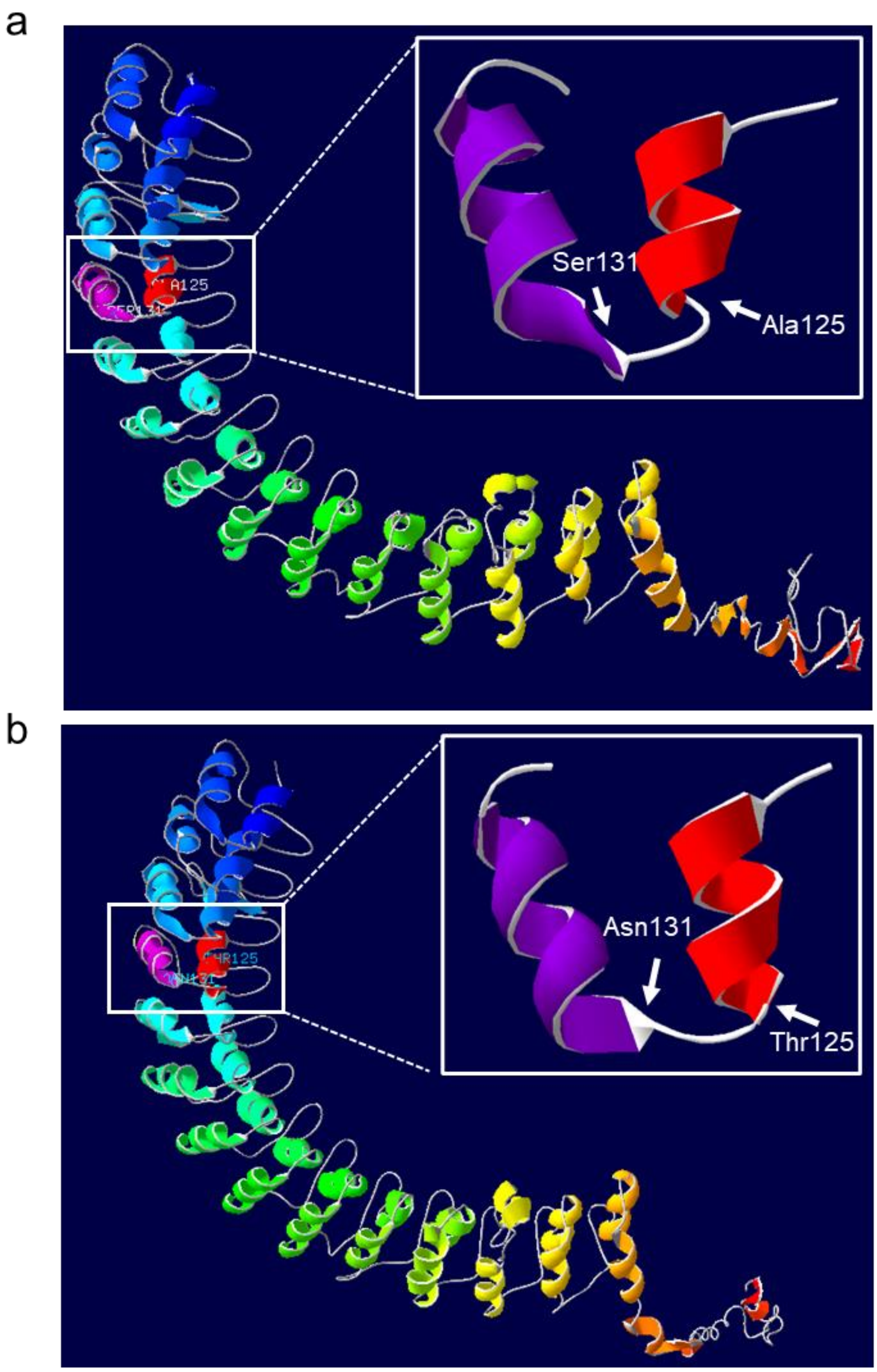

Supplementary Fig. 7. Predicted three-dimensional structures of the TN1 and tn1 proteins.

a–b, Structures of TN1 (**a**) and tn1 protein (**b**) as predicted by I-TASSER (https://zhanglab.ccmb.med.umich.edu/I-TASSER/). The differences around the mutation sites are indicated by the white arrows.

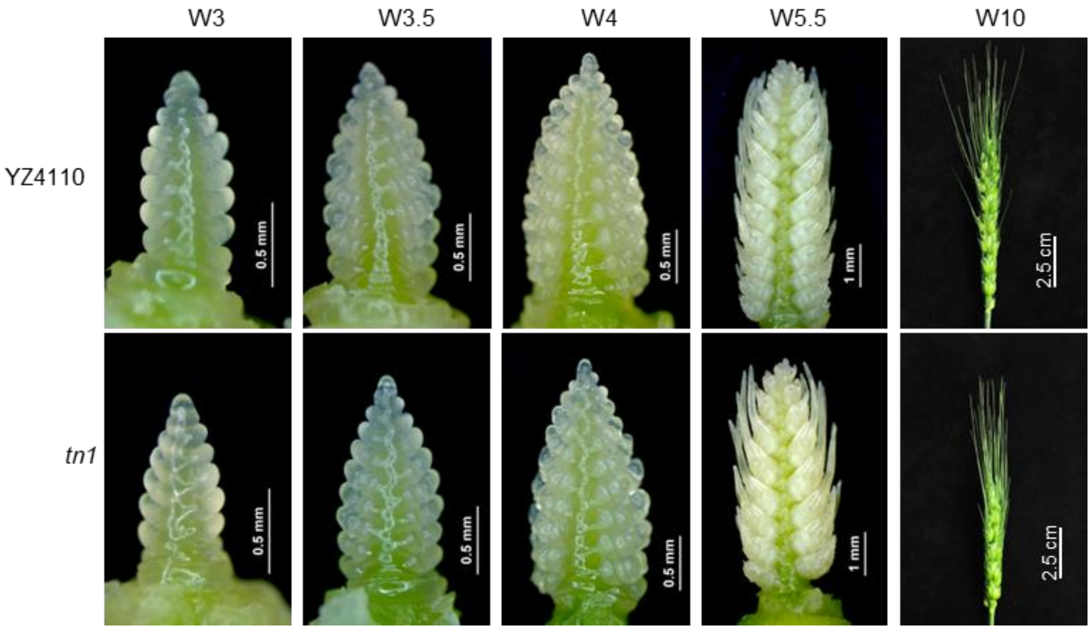

Supplementary Fig. 8. Inflorescence morphologies of YZ4110 and the *tn1* mutant. Inflorescence morphologies of YZ4110 and the *tn1* mutant at different stages. W3, Glume primordium present; W3.5, Floret primordium present; W4, Stamen primordium present; W5.5, Stylar canal closing, ovarian cavity enclosed on all sides but still open above; W10, Styles curved outwards and stigmatic branches spread wide, pollen grains on well-developed stigmatic hairs. The inflorescence developmental stages are based on the Waddington scale [1]. Source data are provided as a Source Data file.

```
TN1-like-6BS   001  ATGGACCCAGCGTTGCATAAGGCGGCGGTTCAAGGTAGCGTAGCAAGCCTGAGGGAGCTGGTGGCCGAGCGTCCCGGCAT
TN1-like-6DS   001  ---------ATGGTTGCACCAGGCGGCGGTGCAGGGGCGCGTGGCAAGCCTGAGGAGGCTGGTGGCCGAGCGCCCCGGCAT
TN1            001  ATGGACCCAGCGTTGCATAAGGCGGCGGTGCAAGGGAGCGTGGCAAGCCTGAGGAAGCTGGTGGCCGAGCGGCCCGGCAT
TN1-like-6BL   001  ATGGATCCAGAGTTGCACAAGGCGGCGGTGCAGGGGAGCGTGACAAGCCTGAGGAAGCTGGTGGCCGAGCGCCCCGGCAT

TN1-like-6BS   081  CCTTGGTTCC---AAGACGCCCCATGGGAACACCGCGCTGCACATCGCCGCCGAGCTCGGCCATGCCGGTTTTGCCGAGG
TN1-like-6DS   073  CCTTGGTTCCTCCAAGACGCCCCAGGAGAACACCGCGCGGC----------CGAGCTCGGCCATGCCGGTTTTGTCGAGG
TN1            081  CCTTGGTTCC---AAGACGCCCCATGGGAACACCGCGCTGCACATCGCGGCCGAAATCGGCCATGCCGGCTTCGCGGAGG
TN1-like-6BL   081  CCTTGGTTCC---AAGACGCCGCAGGGGAACACCGCGCTGCACATCGCCACCGAGCTCGGCCATGCCGGCTTCTTGAAGG

TN1-like-6BS   158  AGGCCCTTGGTGTGGACGACAAGCTGCTCGTCAGCAGGAACGCCGACGGCGACACACCGCTGCACGTGGCGGCTAGGGTG
TN1-like-6DS   143  CGGCCCTTGGTGTGGACGGCAAGCTGCTCGTCAGCAGGAACCACG-CGTGGGCATGGC-CTATG-GAGGCGGCCAGGGCG
TN1            158  AGGTCCTCGGTGCGGACTACAAGCTGCTCGCCACAAGGAACGCCGATGGCGACACGCCGCTGCACCTGGCGGCCAGGGTA
TN1-like-6BL   158  AGGCCCTCGGTGTGGACTGCAAGCTACTCGCCACAAGGAACGCCGACGGCGACACGCCGCTGCACCTGGCGGCCAGGATG

TN1-like-6BS   238  GGGAAGGTGGACGTTGTGGACCTGCTCATCGTCCGAGCATGGGTATGGCATGCGGAGCATCCGCACTACTTCCCCGCCGC
TN1-like-6DS   220  GGGAAGGTGGACGTTGCGGACCTGCTCATCGGCCACGCGTGGGCATGGCCTATGGA------------------------
TN1            238  GGTAAGGTGGACGTGGTGGAGCTGCTACTCAGCCGCGCCAGAGCATGGTCTGCAGAGCAGCCGCA---------------
TN1-like-6BL   238  GGGAAGTTGGACGTGGCGGAGCTGCTCGTCAGCCGCGCCAGCGCATGGCGTGCGGAGCA---------------------

TN1-like-6BS   318  ACAGAATGGACAGACCGGGGAACCTTCCCGGAGCCCCCCTCGCAGCGCGCACGGGCCTCTGTTGATGGCCAACAAGGCCG
TN1-like-6DS   276  ---------------------------------------------CGCGCACGGGCCTCTGTTGATGGCCAACAAGGCCG
TN1            303  ------------------------------GGACTCCCATGGGAGCGAGCAGGGGCCGGTGCTCATGGCCAACAAGGCCG
TN1-like-6BL   297  ---------------------------------CTCCCCTGGGAGCGAGCAGGAGCCTCTGTTCATGGCCAACAAGGCCG

TN1-like-6BS   398  GCGACACCCCGCTCCACGAGGCGGTGAAGCATCGCAGGAGCGCCGTCGCGCTCAGGCTGCTGGAAACCGAGCCAGGCCGC
TN1-like-6DS   311  GCGACACCCCGCTGCACGAGGCGGTGAAGCACGGCAGGAGCGCGGTCGCGCTCAAGCTGGTGGACGCCGAGCCAGGCAGC
TN1            353  GCGACACCCCGCTGCATGAGGCGGTGAAGCACGGCAGGAGCGCCGTGGCGCTCTTGCTGTTGGCCGCCCAGCCCAGCCTA
TN1-like-6BL   344  GCGACACCCCGCTACACGACGCGGTGAAGCAGGGCAGGAGCACCGTGGCGCTCATGCTGTTGGCCGCCGAGCCCAGCGTC

TN1-like-6BS   478  GGCCACGTGCTCAACGTGAAGCAGCAGACGCCGCTACACATCGCCGCGCGGGAGGGCCTCTACGACGTCGTGGCCAAGAT
TN1-like-6DS   391  GGCCACGCGCTCAACGTGAAGCAGCAGACGCCGCTACACATCGCCGCGCGGGAGGGCCTCTACAAAGTCGTCATCAAGAT
TN1            433  GGCCACGCGCTCAACGTGAAGCAGCAGTCGCCGCTGCACATCGCCGCCGGGGAGGGCCTCACCGACGTTGTGGCCAAGAT
TN1-like-6BL   424  GGCCACACGCTCAACATGAGGAAGGAGTCGCCGCTGCACATCGCCGCCCGGGAGGGCCTCGCCGACGTCGTGGCCAAGAT

TN1-like-6BS   558  CGTCAGCCAGCCCTGTGAACACGACAGGTTTTTCCCCTC------CGTCAGCGGCTCCGCCCTGCACCAGGCCGTCCTCA
TN1-like-6DS   471  CAACAGCCAGCCCTGGGTCCACGAGAGCTTTGTCCCCTCTGACTCCGTCAGCGGCTCCGCCCTGCACCAGGCCGTCCTCG
TN1            513  CATCGGCCAGCCCTGGGTCCACGAGAGGTTTGTCCCATCTGACTCCATGAGCAGCACCGCCTTGCACCAGGCCGTCCTCG
TN1-like-6BL   504  CGTCCGCCAGCCCTGGGTCCACGAGAAGTTTGTCCCCTCCGACTCCACCAACGGCACCGCCCTGCACCAGGCCGTCCTCG

TN1-like-6BS   632  GCGGCCACGAACGCGTGGTGGAGATCTTGCTCGACGTGACGCCGCCGGATCAGATCGGGCTGACGGACTCGAGCGAGAAC
TN1-like-6DS   551  GCGGCCACGCACGCGTGGTGGAGATCTTGCTCGACATGACGCCGCGGGAGCAGATCGGGCTGACGGACTCGAGCGAGAAC
TN1            593  GCGGCCACATCCGTGTGGTGGAGATGTTGCTTGACGCGACGCCGCATGAGCAGATCGGGCTGACGGACTCGTCCGAGAAC
TN1-like-6BL   584  GCGGACGCATCCGTATGGTGGAGATGTTGCTTGACGCGACGCCGCAGGAGCAGATCGGGCTGACGGACTCGAGGGAGAAC

TN1-like-6BS   712  AACGCGCTGCACTACGCGGCGCAGAAGAACAACGCCCGCGTGGTGAAGCTGCTGCTCAACCGGAAGGTGGACCTGGCCTA
TN1-like-6DS   631  AACGCGCTGCACTACGCGGCGCAGAAGAACAACGCCCGCGTGGTGAAGCTGCTGCTCAACCGGATGGTGGAGCTGGCCTA
TN1            673  AACGCGCTGCACTACGCGGCGCAGAAGAACAACGGCCGCATGGTGAAGCTGCTGCTGAACCGGAAGATGGACCTGGCCTA
TN1-like-6BL   664  AACGCGCTGCACTACGCGGCGCTCAAGAACAACGCCCGCGTGGTGAGGCTGCTGCTGAACCGGAAGGTGGAGCTGGCCTA

TN1-like-6BS   792  CAAGCGCAACCGTGACCTGCAGTCGCCGCTGCACATGGCCGCCTACTACGGCTCGACCAAGGCCATGGCGGAGCTGCTGC
TN1-like-6DS   711  CAAGCGCAACCGTGACCTGCAGTCCCCGCTGCACGTGGCCGCCTACTACGGGTCGACAAAGGCCATGGCGGAGCTGCTGA
TN1            753  CAAGCGCAACCGCGACCTGCAGTCGCCGCTGCACGTCGCCGCCTCCTACGGCTCGACGGAGGCCATGGTGGAGCTGCTGA
TN1-like-6BL   744  CAAGCGCAACCGCGACCTGCAGTCGCCGCTGCACGTGGCCGCCTGCAAGGGCTCGACGGAGGCCATGGTGGAGCTGCTAA

TN1-like-6BS   872  TGCAGTGCCCTGATGCGGCGGAGATGGTGGACGCCAGCGGCAGGAACGCGTTGCATGTCGCCATCACCATCGGCAAGGTG
TN1-like-6DS   791  AGCAGTGCCCTGACGTGGCGGAGATGGTGGACAGCAACGGCAGGAACGCGTTGCATGTCGCCATCGTCATCGGCAAAGTG
TN1            833  AGCAGTGCCCTGACGTGGCGGAGATGGTGGACAGCAACGGCAGGACAGCTTTCCACGTCGCCGTCACCAGCGGCAAGGTG
TN1-like-6BL   824  GGCATTGCCCTGACGCGGCGGAGACGGTGGACAGCAACGGCAGGAACGCCTTCCGCTTGGCCATCATCAGCGGCAGTGTG

TN1-like-6BS   952  GACGTGCTCCGCTGCCTCCTCAAGCTGGTGCGCCCCGAGGACACTATCAACCATGTCGACCGCGACGGCAACACGCCGCT
TN1-like-6DS   871  GACGTGCTCCGCTGCCTCCTCAAGCACGTGCGCCCGGACGAGATTATCAACAAGGTTGACAACGATGGCAACACAGCGCT
TN1            913  GACGCCCTCAAGTGCCTGCTCAAGCAAGTCCGCCCCGAGGAGATCGTCAATCGCGTCGACCACCAAGGCAACACGCCGCT
TN1-like-6BL   904  GACGCCCTCAAGTGCCTGCTCAAGCACGTCCGCCCCGAGGAGATCGTCAATCATGTCGACCACGCCGGCAACACGCCGCT

TN1-like-6BS   1032 TCATCTGGCGGCCAAGTTTAATCGAGCACCGCTTGGCCTGCTGCTGCTCAAGGACCGCCGGGTGAACCCATGCGTCCTCA
TN1-like-6DS   0951 TCATCTGGCCGCGAAGTTAAATCGAGCTCCGCTTGGCCTGCTGCTGCTCAAGGACCGCAGGGTGAACCCATGCCTCCTCA
TN1            0993 GCACCTAGCCGCTGCGCTGTCGAGCATAAAGTCGGCGCTGCTCCTGCTCAAGGACCGCCGGGTCAGCCCCTGCGTCCTCA
TN1-like-6BL   0984 GCACCTCGCGGCTGCCCTGTCGCGCATCCAGTCGACGCTGCTCCTGCTCAAGGACCGCCGCGTCAACCCCTGCGTCCTCA

TN1-like-6BS   1112 ACCGCAACGGCCGCACCGCTCGGAGCTTTATCGAGACACA-AACACCAGTGGATAACCTCACGGTAATTTATGTCTACTT
TN1-like-6DS   1031 ACAGGGAAGGCCGCACCGCACGGAGCATTACCGAGGAGCA-AACACCTGTCGATAGCTTCACGTTATTCCAGGTCTACTT
TN1            1073 ACCGGGACGGCCAGTCCGCGCGCAGTCTCATCGAGAAGCGCGCCACCACTGAGGAGTTGGACACCTA-CGAGATGTACCT
TN1-like-6BL   1064 ACCGGGACGGCCAGTCTGCGCGCAGTCTCCTAGAGAAGCGCGACACCACCGAGGAGATGGACGCCTA-CGAGATGTACAT
```

Supplementary Fig.9 Continued

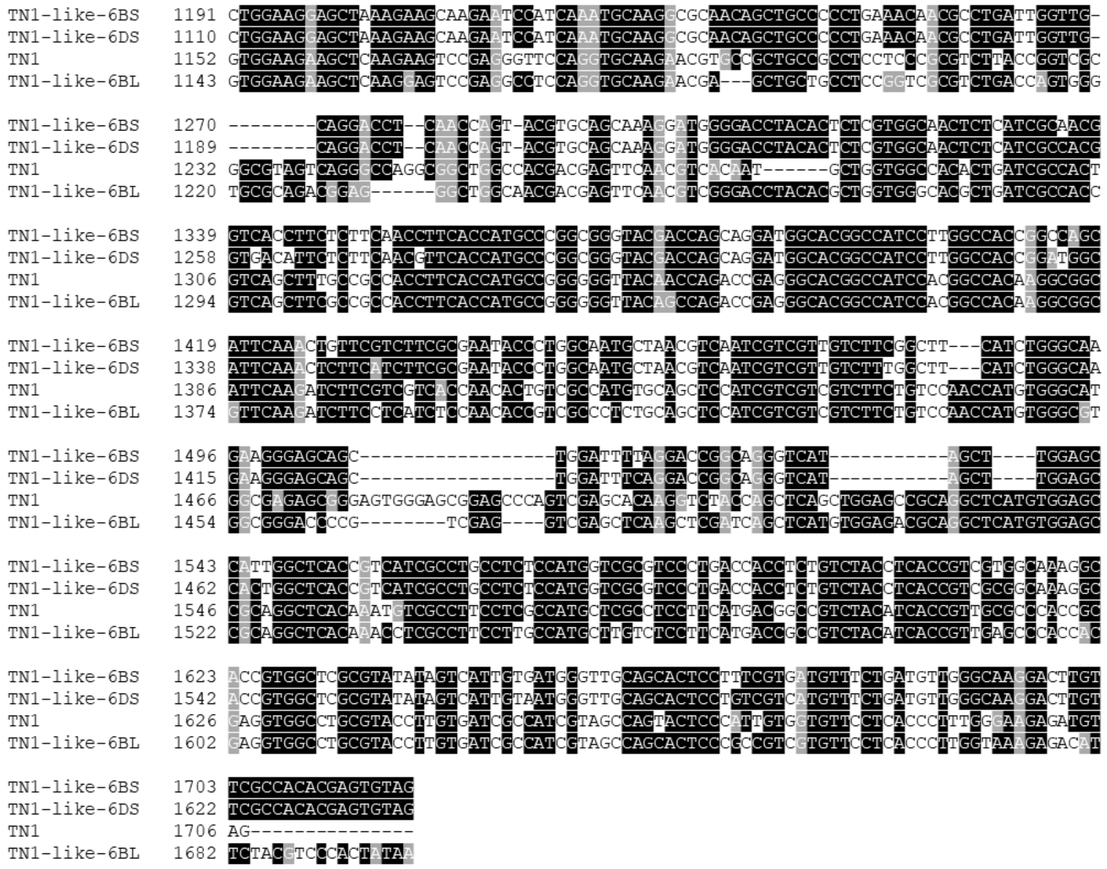

Supplementary Fig. 9. Alignment of the coding sequences of *TN1*, *TN1-like-6BS*, *TN1-like-6BL*, and *TN1-like-6DS*.

The mutation sites in *TN1* are highlighted in red. White, gray, and black backgrounds represent the extent of sequence identity. *TN1*, *TraesCS6B02G013100*; *TN1-like-6BL*, *TraesCS6B02G437600*; *TN1-like-6BS*, *TraesCS6B02G013000*; *TN1-like-6DS*, *TraesCS6D02G011400*.

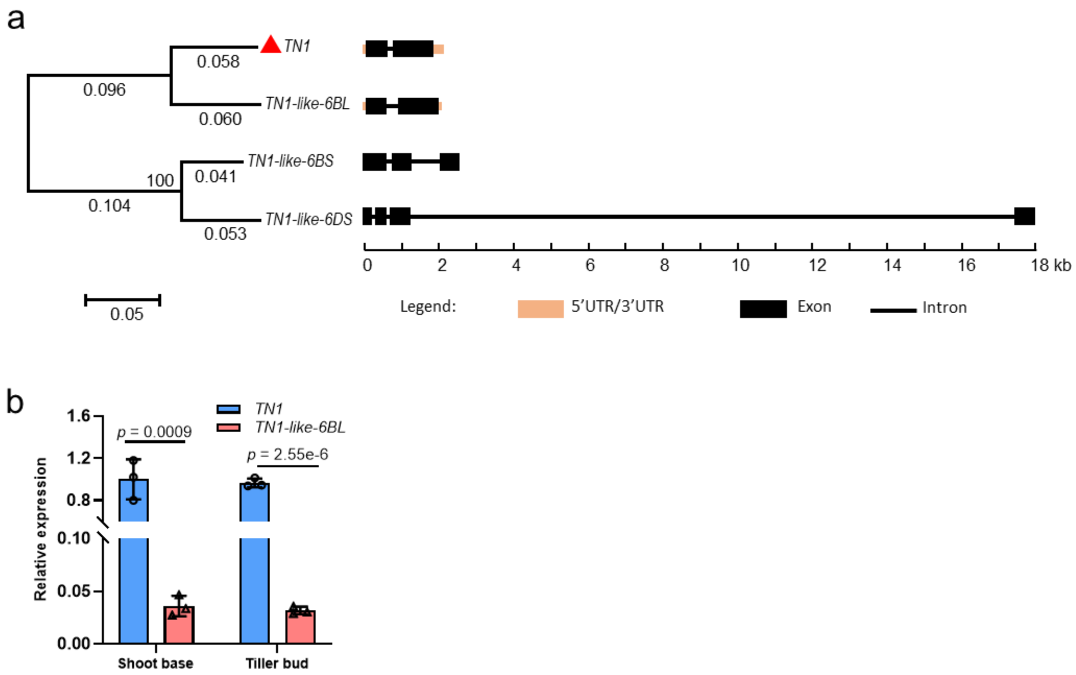

Supplementary Fig. 10. Phylogenetic tree, gene structures, and expression pattern of *TN1* homologs in hexaploid wheat.

a, Three *TN1* homologs were identified by performing a BLAST search using the complete coding sequence of *TN1* as a query. The phylogenetic tree was constructed using MEGA 7.0 and the neighbor-joining method. Bootstrap values calculated from 1,000 replicates are marked on the branch nodes; branch length represents substitutions per site and is marked above the branches. The *TN1* gene is indicated by the red triangle.

b, Relative expression levels of *TN1* homologs at the shoot base and in tiller buds. The relative expression levels were normalized to *TaGAPDH*. Data are means ± SD of three biological replicates, and *p* values are indicated by two-tailed unpaired *t*-test. Source data underlying Supplementary Fig. 10b are provided as a Source Data file.

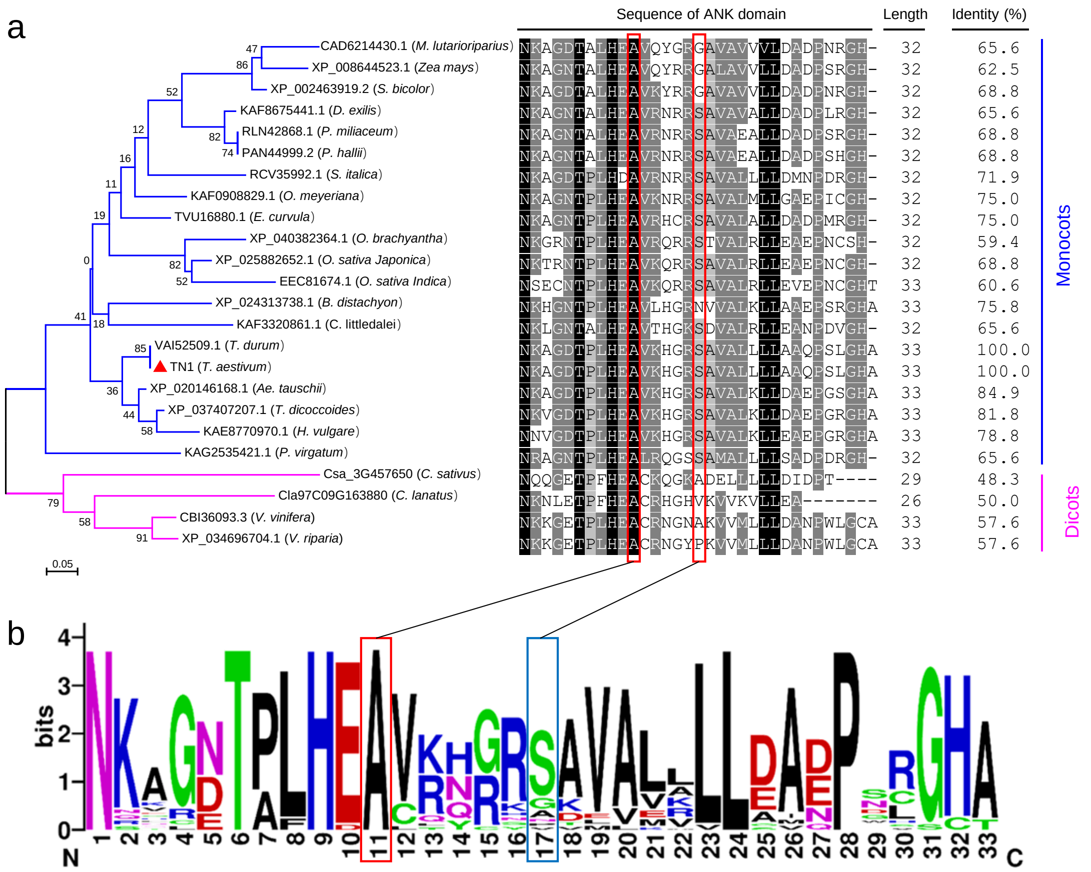

Supplementary Fig. 11. Phylogenetic analysis for the third ANK (ANK^{3rd}) domain of TN1.

a, Phylogenetic tree of the ANK^{3rd} domain in TN1 and its homologs from monocots and dicots. The phylogenetic tree was constructed using MEGA 7.0 and the neighbor-joining method, and bootstrap values calculated from 1,000 replicates are marked on the branch nodes. Branch length represents substitutions per site. The sequences of the ANK domain used for phylogenetic tree construction are listed on the right panel. The mutation sites in the tn1 protein are indicated with red boxes. **b,** Conservation analysis of the ANK^{3rd} domain in TN1. The red/blue-boxed regions represent the positions of the mutation sites.

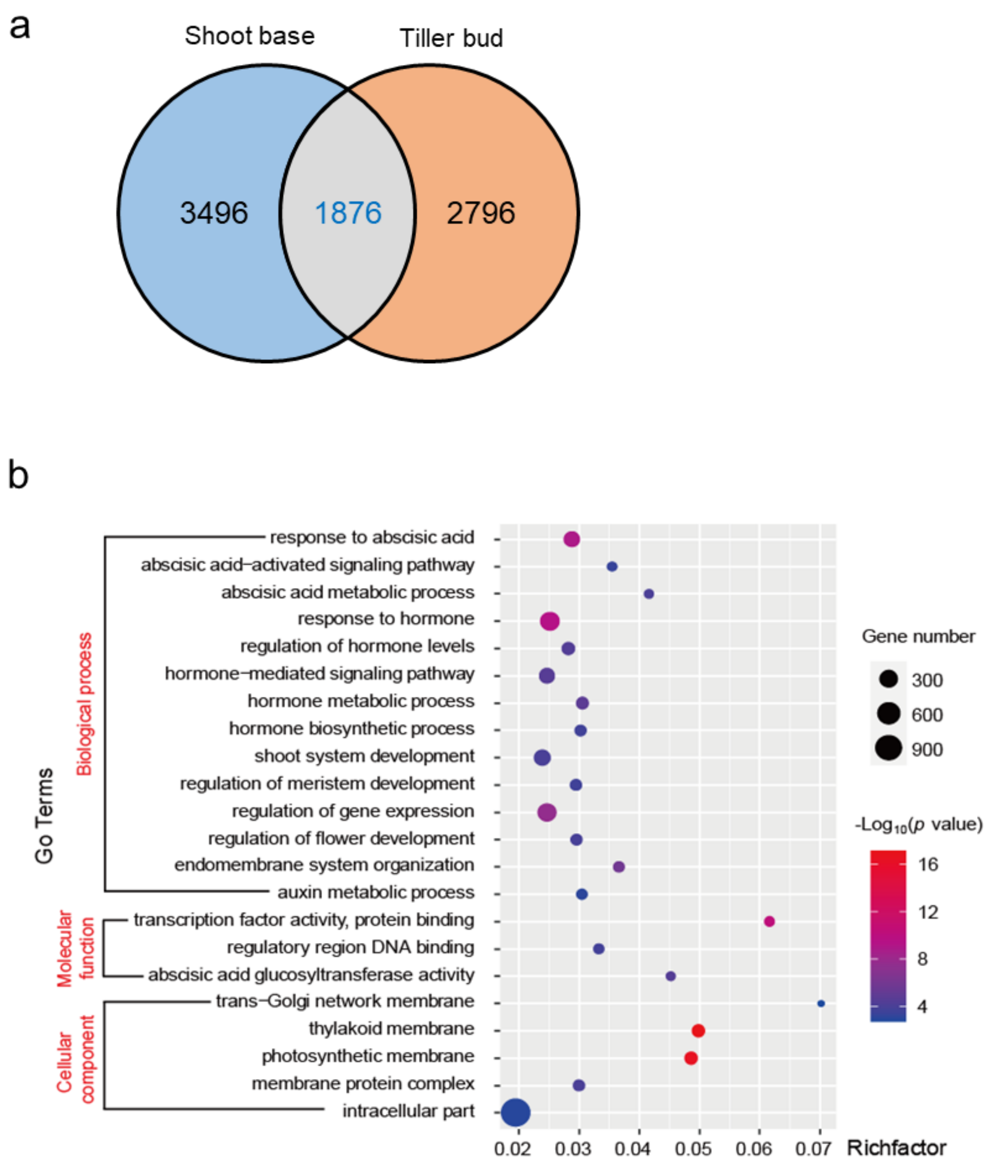

Supplementary Fig. 12. RNA-seq analysis between YZ4110 and the *tn1* mutant. **a,** Venn diagram of differentially expressed genes (DEGs) between YZ4110 and the *tn1* mutant at the shoot base and in tiller buds. Genes with $\log_2$(fold-change) ≥ 1, *p* value < 0.05 were considered as DEGs. **b,** Major pathways of DEGs in Gene Ontology (GO) analysis. The unadjusted *p* values in (**a**) and (**b**) were determined by two-sided Fisher's exact test, no adjustment made for multiple comparisons.

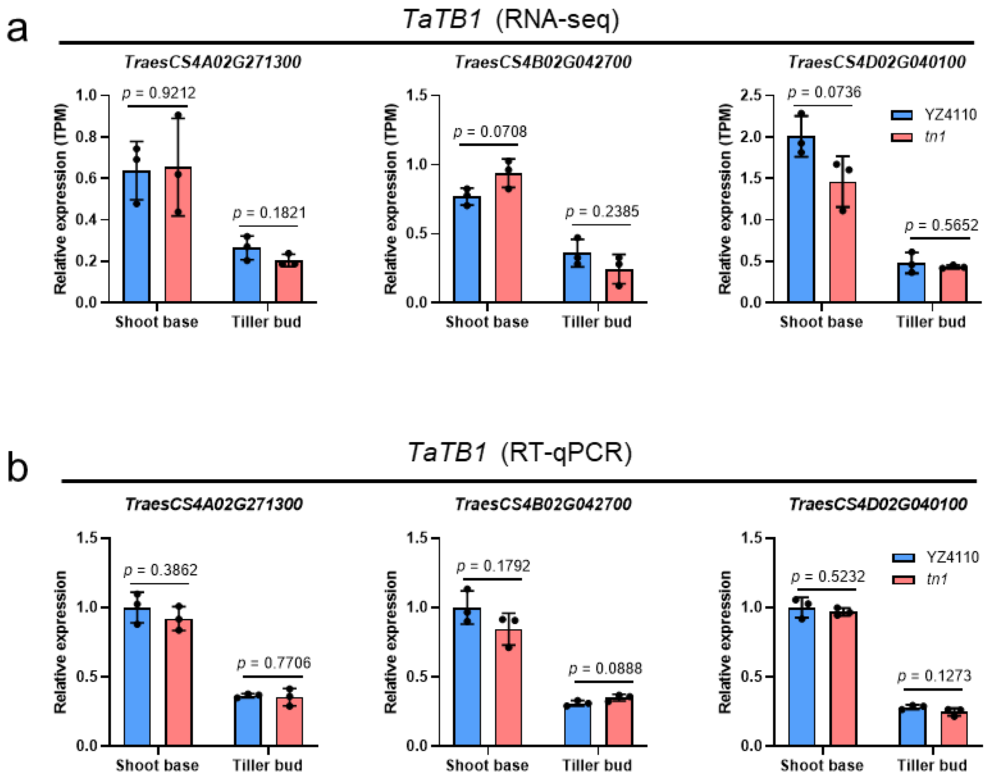

Supplementary Fig. 13. Expression patterns of *TaTB1* genes in the *tn1* mutant. **a–b,** Expression analysis of *TaTB1* genes based on RNA-seq data (**a**) and RT-qPCR (**b**). The expression levels in RNA-seq are represented as transcripts per million (TPM). The relative expression levels were normalized to *TaGAPDH*. Data are means ± SD of three biological replicates, and p values are indicated by two-tailed unpaired t-test. Source data are provided as a Source Data file.

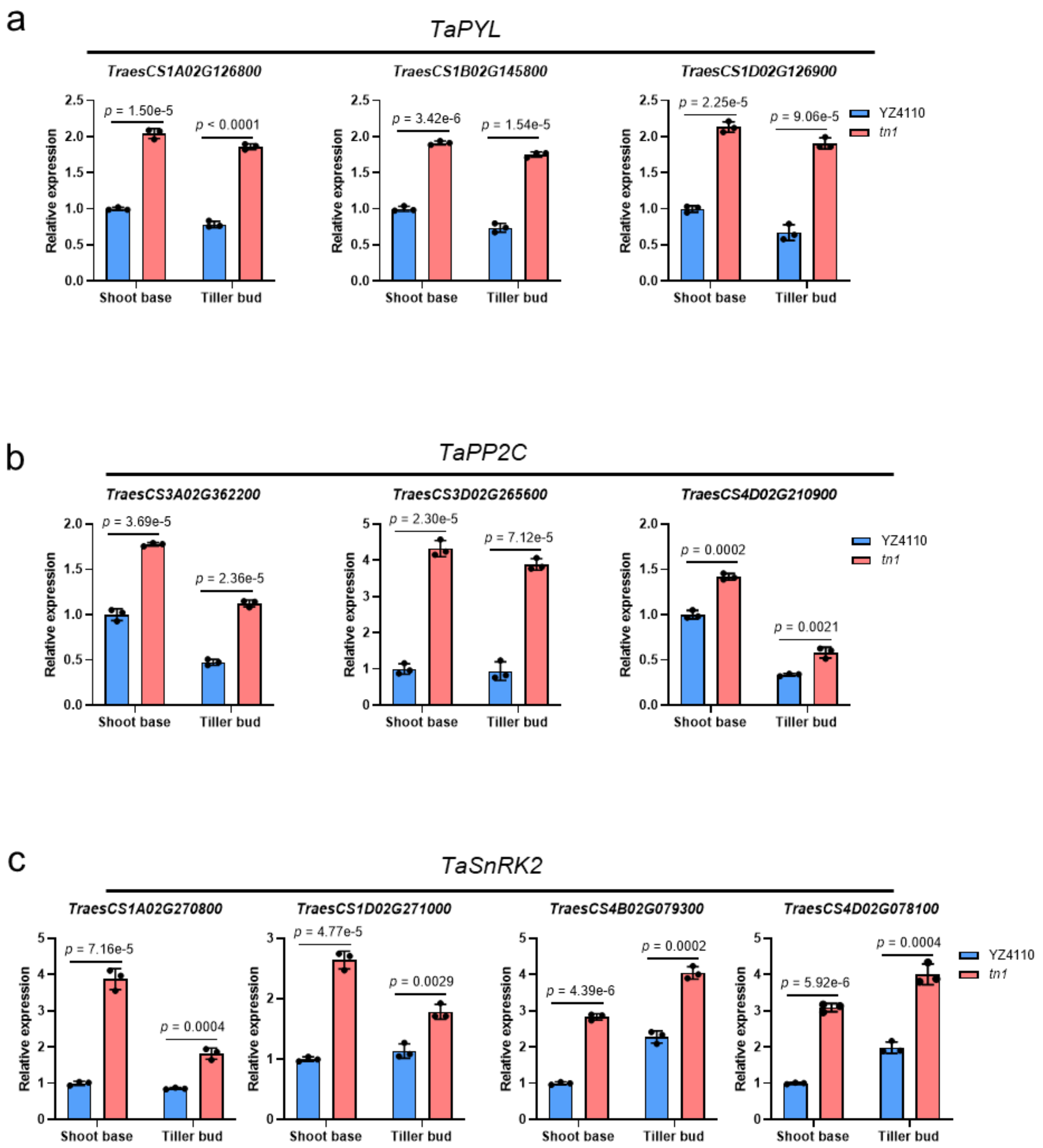

Supplementary Fig. 14. Expression patterns of abscisic acid (ABA)–related genes in the *tn1* mutant.

a–c, Expression patterns of the ABA signaling genes *TaPYL* (**a**), *TaPP2C* (**b**), and *TaSnRK2* genes (**c**) in YZ4110 and the *tn1* mutant. The relative expression levels were normalized to *TaGAPDH*. Data are means ± SD of three biological replicates, and *p* values are indicated by two-tailed unpaired *t*-test. Source data are provided as a Source Data file.

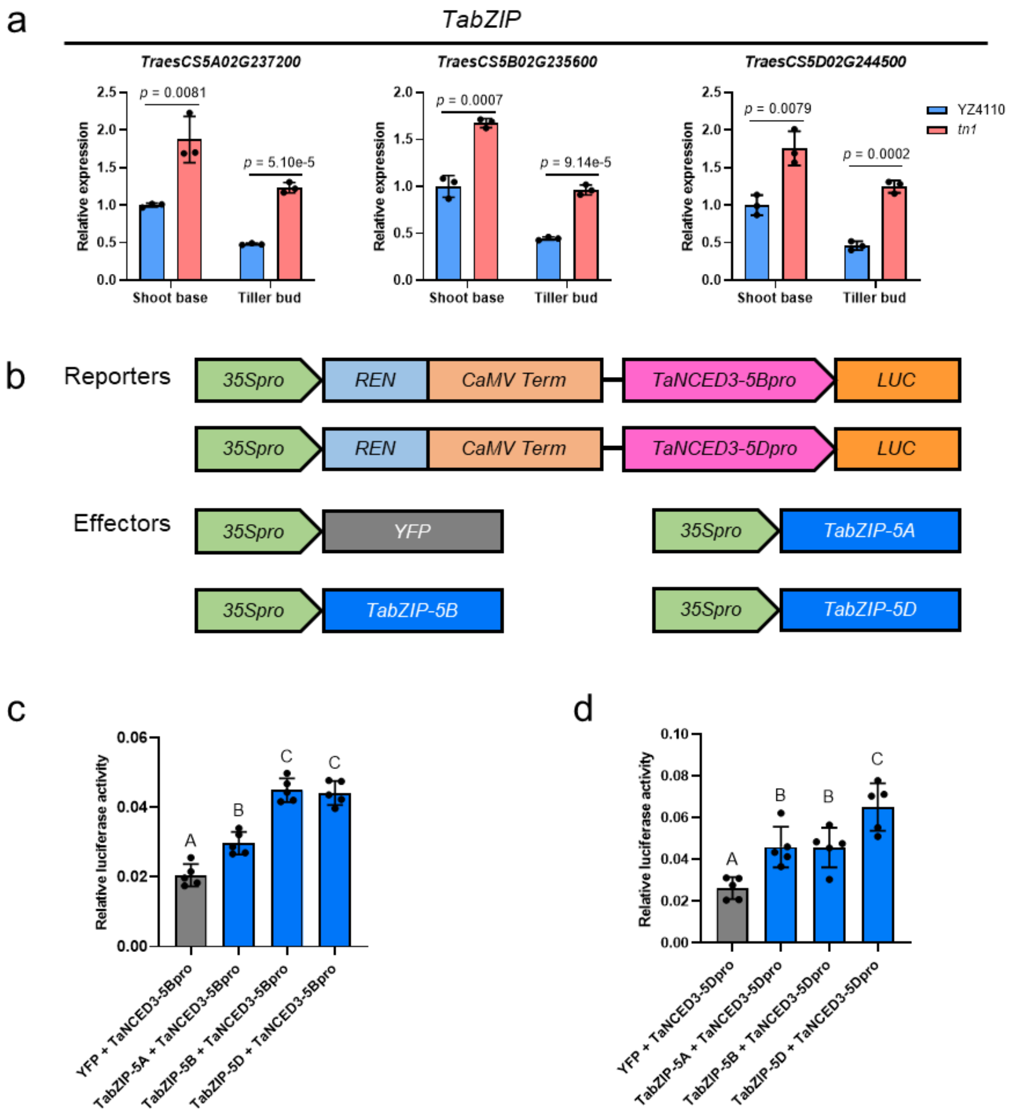

Supplementary Fig. 15. TabZIP proteins can activate transcription from the *TaNCED3* promoters in a transient transactivation assay.

a, RT-qPCR analyses showing the expression patterns of *TabZIP-5A/5B/5D* genes in the *tn1* mutant. The relative expression levels were normalized to *TaGAPDH*. Data are means ± SD of three biological replicates, and p values are indicated by two-tailed unpaired t-test. **b,** Schematic diagrams of the effector and reporter constructs used in the dual-luciferase reporter assays. *35Spro*, cauliflower mosaic virus (CaMV) 35S promoter; *CaMV term*, CaMV terminator; *REN*, *Renilla* luciferase gene; *LUC*, firefly luciferase gene. **c–d,** Transactivation of *TaNCED3-5B/5D* genes by TabZIP proteins. The relative luciferase activity is expressed as the LUC/REN activity ratio. Data in (**c**) and (**d**) are shown as means ± SD (n = 5 biologically replicates). Different letters indicate significant differences between groups, as determined by one way ANOVA with Duncan's multiple range tests (p < 0.01). Source data are provided as a Source Data file.

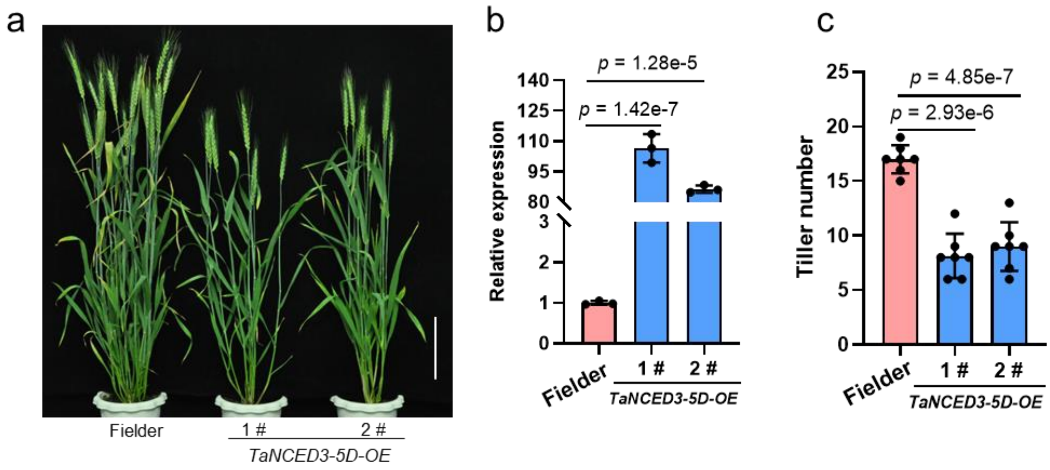

Supplementary Fig. 16. Morphological characteristics of *TaNCED3-5D* overexpression wheat plants.

a, Morphology of T_1 generation *TaNCED3-5D* overexpression wheat plants. Scale bar, 15 cm. **b**, Relative expression levels of *TaNCED3-5D* in the leaf of transgenic lines. The relative expression levels were normalized to *TaGAPDH*. Data are means ± SD of three biological replicates. **c,** Tiller number for Fielder, and positive transgenic lines. Data are means ± SD (n = 7 independent transgenic plants). The p values in (b) and (c) are indicated by two-tailed unpaired t-test. Source data are provided as a Source Data file.

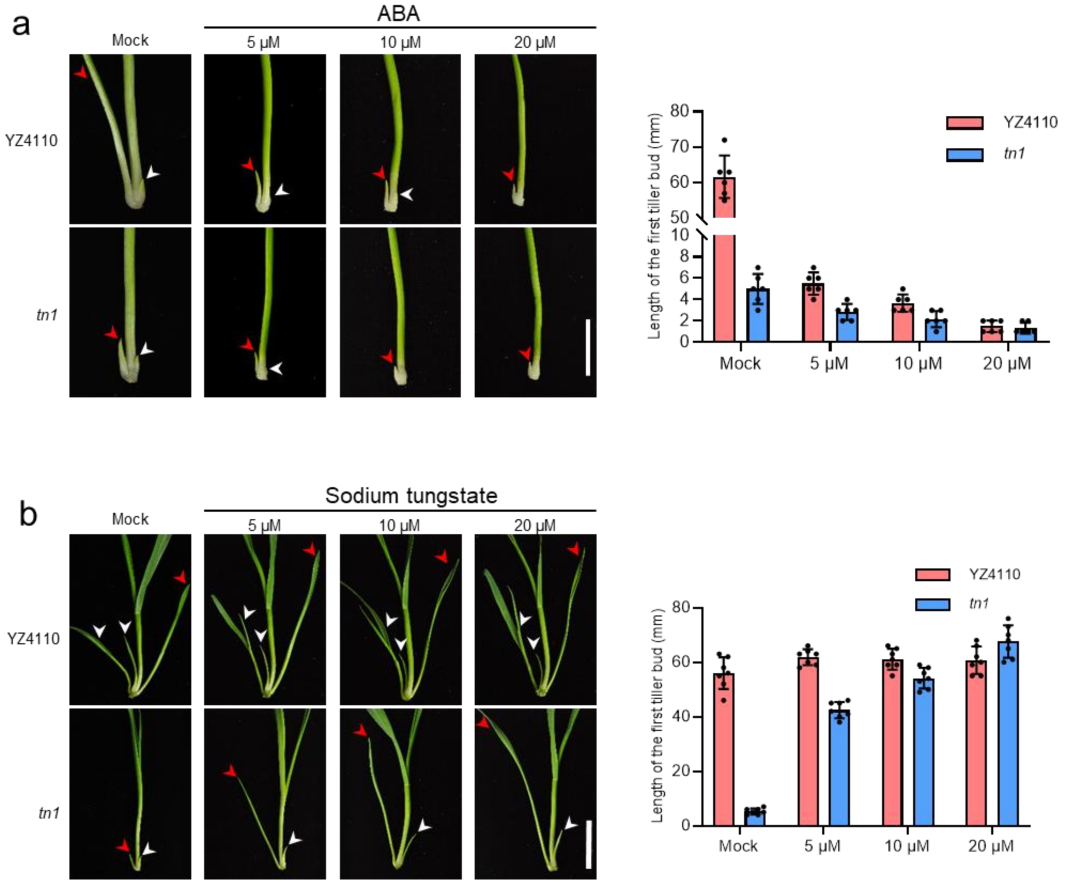

Supplementary Fig. 17. Effects of ABA and sodium tungstate treatments on tiller bud outgrowth in YZ4110 and the *tn1* mutant.

a, ABA application inhibits tiller bud outgrowth. $n = 6$ independent plants for each treatment. **b,** The ABA biosynthesis inhibitor sodium tungstate partially rescues the low-tillering phenotype of the *tn1* mutant. $n = 7$ independent plants for each treatment. Lengths of the first tiller buds (red arrows indicated) in (**a**) and (**b**) were measured. The white and red arrows indicate the presence of tiller buds. Data are means ± SD. Scale bars, 1 cm in (**a**), 2 cm in (**b**). Source data are provided as a Source Data file.

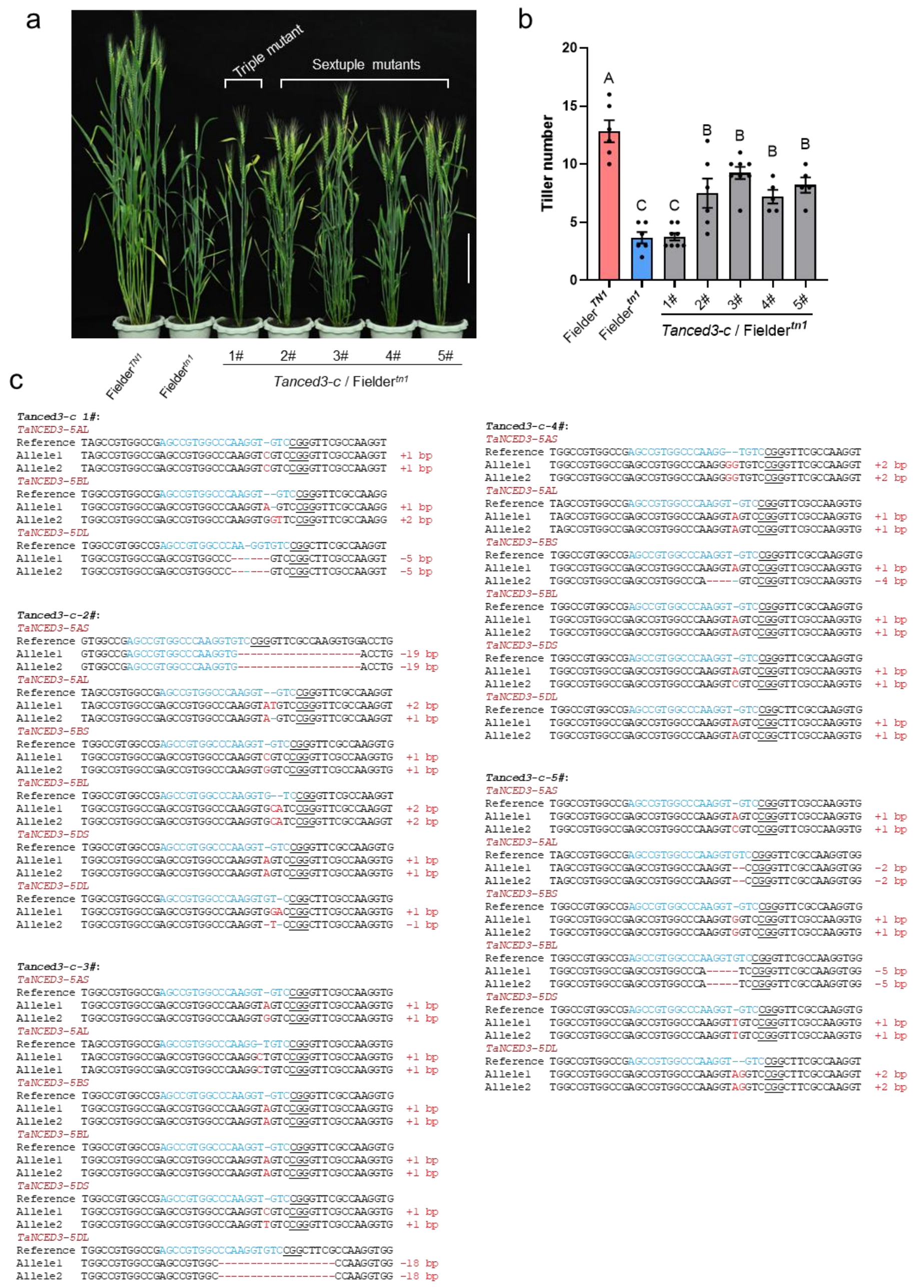

Supplementary Fig. 18. Phenotypes of *Tanced3* mutants generated by CRISPR/Cas9 in the Fielder^{tn1} background.

a, b Representative phenotypes (**a**) and tiller number (**b**) of the *tn1* mutants generated by CRISPR/Cas9-mediated gene editing. The tiller numbers were measured from the T_1 generation transgenic lines at grain-filling stage. Scale bar, 15 cm in (**a**). Data in (**b**) are means ± SEM, Tiller numbers of Fielderr^{TN1} ($n = 6$), Fielderr^{tn1} ($n = 6$) and *Tanced3* mutants (n = 8/6/8/5/5) were measured at grain-filling stage from independent transgenic plants. Different letters indicate significant differences between groups, as determined by one way ANOVA with Duncan's multiple range tests ($p < 0.01$). **c** Genotype of the mutant lines. The target sites are marked in blue, and the PAM is underlined. The red letters indicate nucleotide insertions, and the red dashed lines represent nucleotide deletions. Source data underlying Supplementary Fig. 18a, b are provided as a Source Data file.

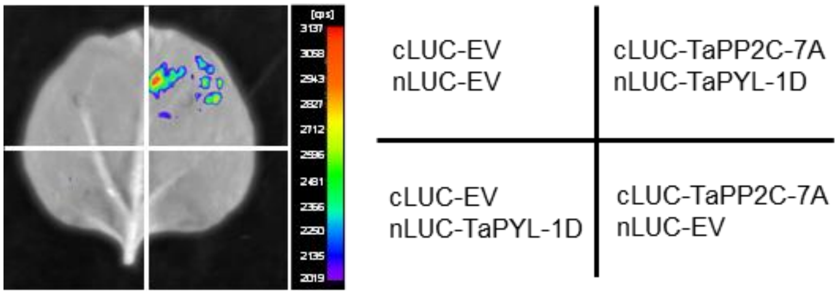

Supplementary Fig. 19. Interaction between TaPP2C-7A and TaPYL-1D revealed by LCI assay.

Luciferase complementation imaging (LCI) assays showing that TaPP2C-7A physically interacts with TaPYL-1D in *N. benthamiana* leaves. Source data are provided as a Source Data file.

Supplementary Table 1. Genetic analysis of the *tn1* mutant.

Cross combination	F_1		F_2		Ratio	χ^2 (3:1)	*p* value†
	Wild type	*tn1*	Wild type	*tn1*			
YZ4110 / *tn1*	5	0	315	116	2.72:1	0.842	0.3588
JM20 / *tn1*	46	0	8203	2952	2.78:1	12.7419	0.0004

† The *p* values were determined by Chi-square (χ2) goodness-of-fit tests, df = 1.

Supplementary Table 2. Candidate genes predicted within the 352-kb region.

GENE_ID	Location	Uniprot ID	FunctionalAnnotation_v1
TraesCS6B02G012800	6B:8045847-8049871	A0A3B6PFP1	Myb family transcription factor-like protein
TraesCS6B02G012900	6B:8123320-8129270	A0A3B6PEA4	Male sterility protein
TraesCS6B02G015600LC	6B:8134089-8134577	No BLAST hits	Amiloride sensitive sodium channel subunit gamma
TraesCS6B02G015700LC	6B:8136084-8136398	No BLAST hits	CREA_PSEPU Creatinase
TraesCS6B02G013000	6B:8138326-8140897	A0A3B6PEF5	Ankyrin repeat family protein
**TraesCS6B02G013100**	**6B:8234408-8236549**	**A0A3B6PD15**	**Ankyrin repeat family protein**
TraesCS6B02G015800LC	6B:8244047-8244994	No BLAST hits	RNA directed DNA polymerase
TraesCS6B02G015900LC	6B:8320567-8321582	No BLAST hits	Retrovirus related Pol polyprotein from transposon
TraesCS6B02G016000LC	6B:8341233-8342490	No BLAST hits	Calmodulin binding transcription activator
TraesCS6B02G016100LC	6B:8370169-8371427	No BLAST hits	Calmodulin binding transcription activator
TraesCS6B02G013200	6B:8372877-8375684	A0A3B6PG38	Disease resistance protein (NBS-LRR CLASS) FAMILY
TraesCS6B02G013300	6B:8385071-8389993	A0A3B6PFP6	Disease resistance protein (NBS-LRR CLASS) FAMILY

Supplementary reference

[1] Waddington, S.R., Cartwright, P.M. & Wall, P.C. A quantitative scale of spike initial and pistil development in barley and wheat. *Ann. Bot.* **51**, 119-130 (1983).
