# Supplementary material for: Tiller Number1 encodes an ankyrin repeat protein that controls tillering in bread wheat
Source: Nat Commun. 2023 Feb 14;14:836. doi: 10.1038/s41467-023-36271-z (PMC9929037; doi:10.1038/s41467-023-36271-z)
Supplement: Supplementary file 3 — Description of Additional Supplementary Files [file 41467_2023_36271_MOESM3_ESM.pdf]

Descriptions of Additional Supplementary Files

File Name: Supplementary Data 1

Description: Primers used for gene mapping.

File Name: Supplementary Data 2

Description: Primers used for amplification of the predicted genes in the candidate region.

File Name: Supplementary Data 3

Description: ANK domain sequences used for phylogenetic analysis.

File Name: Supplementary Data 4

Description: List of the 47 common wheat cultivars used for haplotype analysis of *TN1*.

File Name: Supplementary Data 5

Description: List of differentially expressed genes (DEGs) in the shoot base of YZ4110 and the *tn1* mutant.

File Name: Supplementary Data 6

Description: List of differentially expressed genes (DEGs) in the tiller bud of YZ4110 and the *tn1* mutant.

File Name: Supplementary Data 7

Description: List of Gene Ontology (GO) terms of differentially expressed genes (DEGs) between YZ4110 and the *tn1* mutant.

File Name: Supplementary Data 8

Description: Primers used for *TN1* cloning, vector construction, expression analysis, and *in situ* hybridization.
